# Jumping and leaping estimations using optic flow

**DOI:** 10.3758/s13423-024-02459-7

**Published:** 2024-01-29

**Authors:** Lisa P. Y. Lin, Sally A. Linkenauger

**Affiliations:** 1 Department of General Psychology, Justus-Liebig University Gießen, Gießen, Germany; 2 Department of Psychology, Lancaster University, Lancaster, UK

**Keywords:** Perception and action, Optic flow, Perceptual-motor calibration

## Abstract

Optic flow provides information on movement direction and speed during locomotion. Changing the relationship between optic flow and walking speed via training has been shown to influence subsequent distance and hill steepness estimations. Previous research has shown that experience with slow optic flow at a given walking speed was associated with increased effort and distance overestimation in comparison to experiencing with fast optic flow at the same walking speed. Here, we investigated whether exposure to different optic flow speeds relative to gait influences perceptions of leaping and jumping ability. Participants estimated their maximum leaping and jumping ability after exposure to either fast or moderate optic flow at the same walking speed. Those calibrated to fast optic flow estimated farther leaping and jumping abilities than those calibrated to moderate optic flow. Findings suggest that recalibration between optic flow and walking speed may specify an action boundary when calibrated or scaled to actions such as leaping, and possibly, the manipulation of optic flow speed has resulted in a change in the associated anticipated effort for walking a prescribed distance, which in turn influence one’s perceived action capabilities for jumping and leaping.

Visual perception is an active and continuous process, whereby we use the changes in optical information over time to specify potential opportunities for actions available and to aid navigation in the environment. To perform actions adaptively and avoid performance errors, the perceiver must be able to perceive those behaviourally relevant properties in the environment relative to their action capabilities (Chemero, 2003; Gibson, 1979). Warren (1984) assessed individuals’ ability to perceive the optimal/maximum height of steps that affords bipedal climbing and found that independent of body height, tall and short individuals perceived the maximum step height that affords bipedal climbing to be approximately 0.88 of leg lengths. This finding demonstrated that individuals are calibrated to their action capabilities and use the task-relevant part of their bodies—that is, their leg length—to scale possibilities for action and to distinguish between possible and impossible actions.

However, one’s action capabilities are not completely stagnant, and humans must make motor decisions under changing and at times unpredictable environmental conditions, rendering it necessary for the scaling between perceptual information and action to be recalibrated for actions to be executed adaptively and to avoid motor errors (Brand & de Oliveira, 2017; van Andel et al., 2017). Mark (1987) had participants make judgments about their bipedal stair climbing capabilities while standing on blocks. Information about the climb-on-ability of steps is scaled to one’s body dimensions such as leg length and standing eye height (Warren, 1984). By standing on the blocks, participants’ eye-height information for stair height scaling was increased and the same stair was of a smaller proportion of their new standing eye-height, and this information must be recalibrated to allow accurate perception of action possibilities. Results demonstrated that even without practicing the specific action (i.e., stair climbing), participants’ subsequent affordance judgments were consistent with the changes in body dimensions (i.e., eye height), suggesting that individuals could rapidly relearn their action capabilities after a small amount of exploratory behaviour and recalibrated to changes in their eye height. Hence, the recalibration between perceptual information and action is fundamental to the successful performance of visually guided actions.

For walking, idiothetic information—which consists of muscular and joint proprioception, motor efference, and vestibular information—contributed to human perception of self-motion, and this internal sense of self-motion covaried with the visual information specifying the displacement in the environment resulting from the movement of the perceiver, known as global optic flow (Gibson, 1950; Rieser et al., 1995). Optic flow can be defined as the transformation in the pattern of the visual array resulting from the movement of the perceiver. Global optic flow is generated as the perceiver moves through the environment, and over time, through action, the rate of optic flow is calibrated with idiothetic information about the locomotor distance travelled by gait, and the resulting visual-locomotor coupling allows perceivers to guide their actions, and regulate their gait transition, to determine speed and heading (Gibson, 1950; Mohler et al., 2007; Warren & Hannon, 1990; Warren et al., 2001). Additionally, calibration between movement and optic flow allows the perceiver to determine the locomotor distance traversed by integrating the rate of optic flow relative to gait, such that they can determine the locomotor distance travelled from optic flow by knowing one’s gait, to estimate travel distance from optic flow, given that appropriate scaling information is provided (Frenz & Lappe, 2005; Frenz et al., 2003; Redlick et al., 2001), and to accurately estimate the amount of walking required to traverse a given extent, even in the absence of continuous visual information (Rieser et al., 1990).

Optic flow calibration is fundamental to visually controlled locomotion, and studies have found evidence that humans can quickly and flexibly adapt to perturbations in this learned relationship between gait and optic flow rate. Rieser et al. (1995) introduced a discrepancy between walking and optic flow by having participants walk on a treadmill that was being pulled by a tractor moving at a speed that was different from the participants’ walking speed. After experiencing optic flow speed that was slower than their walking speed, participants overshoot the target when asked to walk blindly towards a previously seen location. Conversely, after experiencing optic flow speed that was faster, participants tended to undershoot the target. These results demonstrated that the perception of the amount of walking required to traverse a prescribed distance can be altered by the recalibration of optic flow relative to gaits, thus influencing participants’ subsequent blind-walking judgments. This recalibration of optic flow relative to gait could be generalized to subsequent blind-walking and blind side-stepping but failed to generalize to blind-throwing /turning-in-place. These findings led Rieser et al. (1995) to postulate that the calibration of locomotion is functionally organized, and calibration of one action can be transferred to other functionally similar actions. Similarly, Withagen and Michaels (2002) found generalization of optic flow calibration from walking to crawling; Kunz et al. (2009) found generalization from walking to imagining walking. Similarly, recent studies using various forms of immersive displays (e.g., CAVEs, VR) have reported similar generalization of optic flow calibration to subsequent walking (Adams et al., 2018; Mohler et al., 2004, 2007; Solini et al., 2019, 2021; Waller & Richardson, 2008; Ziemer et al., 2013).

So, if one adapted to a change in the optical distance that accompanies each step, does this learning translate to other types of locomotor actions in far space that are less kinematically similar to walking, such as leaping and jumping? Studies on optic flow calibration reported that the recalibration of optic flow relative to gait could be transferred to actions that serve the same locomotor function as walking, such as crawling, but not to functionally dissimilar actions, such as throwing or kicking (Bruggeman and Warren, 2010; Rieser et al., 1995; Withagen & Michaels, 2002). Although the transfer of optic calibration has been observed in functionally similar actions, not all evidence is consistent in showing the transfer of calibration between functionally similar actions. For example, optic flow calibration relative to gait failed to transfer to other locomotor actions performed by a different limb (Durgin et al., 2003) or only weakly transferred to actions that have similar functions but are generally less well practiced (Durgin et al., 2004; Kunz et al., 2013; Rieser et al., 1995). For example, Kunz et al. (2013) found that optic flow calibration relative to walking only weakly influences subsequent wheelchair-wheeling in novice wheelchair users.

Beyond optic flow calibrations, studies examining the calibration transfer between different functionally similar actions in far space have found that calibration does not always transfer. For instance, Day et al. (2015) examined the transfer of calibration between leaping and stepping and reported that the calibration between stepping and leaping is unidirectional. In particular, practicing maximum leaping transfers to the perception of maximum stepping. However, practicing stepping did not transfer to the perception of maximum leaping distance. This finding suggests that while the two actions are functionally similar, the calibration transfer was not bidirectional. In a study conducted by Franchak (2020), the calibration between fitting and squeezing through doorways was investigated. These two actions differed in a critical aspect: whether participants were permitted to make contact with the sides of the doorway. For the squeezing task, participants were required to navigate through a doorway while wearing a backpack. In this task, contact with the doorway was permitted and a “failure” was defined as instances where participants became stuck and could not successfully pass through the doorway. For the fitting task, participants and their backpacks were not allowed to make contact with the sides of the doorway during the passage. A “failure” in this context referred to instances where participants’ bodies or backpacks made contact with either side of the doorway. In this study, they found that despite the functional similarity (i.e., both involved passing through doorways of varying widths), calibration failed to transfer between the two actions. This led the author to postulate that calibration transfers depend on both functional and informational similarity. In other words, calibration transfers occur between actions that are functionally similar and rely on the same perceptual information for detection or scaling. Hence, one could reasonably postulate that the recalibration of optic flow relative to gait may influence one’s perceived ability to perform a certain type of launching action but not the others.

We examined the effect of optic flow calibration relative to gait on perceived jumping ability and leaping ability. We have opted to examine two different types of gaits—leaping, which is a highly familiar action and kinematically similar to walking in terms of leg oscillation, and two-footed horizontal jumping, which has different coordinative movement patterns to walking and the performance outcomes tend to be more variable. Furthermore, it is not a common action in everyday lives, and previous studies have shown that optic flow recalibration to walking would result in no/weak generalization to functionally similar actions that are not as well practiced (Kunz et al., 2013).

In this study, we manipulated the perceptual-motor coupling between walking and optic flow by having participants walk on the treadmill at the same speed while experiencing either moderate or fast optic flow. We expected that following recalibration to either fast or moderate optic flow relative to gait would influence participants’ judgments of their leaping ability due to its kinematic similarity to walking but would not or only weakly influence their two-footed jumping ability as it involves distinct coordinative movement patterns compared with walking.

## Experiment 1

In this experiment, we investigated the effect of optic flow speed on the perception of action boundary for leaping. Participants walked on the treadmill while wearing a head-mounted display that provided them with either fast or moderate optic flow. Following calibration in the virtual environment, participants estimated their maximum leaping ability in a real-world environment.

### Method

#### Participants

G*Power software application (Faul et al., 2007) was used to perform an a priori power analysis to estimate sample sizes required to achieve adequate power. The required power was set at 1− β = .85, and the level of significance was kept at α = .05.

We expected a large effect size of *f* = .4 based on Linkenauger and Readman (2020), where a similar virtual reality programme was used, and participants were asked to make slant estimations following experience forward walking with either fast or moderate optic flow. In this study, an *f* value of .72 was obtained using a sample size of *N* = 15. Power analysis indicated that a sample size of *N* = 6 would be sufficient to achieve a power of .85 and an alpha of .05. However, due to the slight difference in methodology between the current study and that of Linkenauger and Readman (2020), we doubled the number of participants recruited by Linkenauger and Readman (2020) and increased our sample to 30 for both experiments to ensure we had sufficient power and due to the possibility of technical failure with this type of equipment.

Thirty participants (five males) between 18 and 22 years of age (*M*_age_ = 20.21 years, *SD*_age_ = 1.03) were recruited from Lancaster University through opportunity sampling. One participant was excluded from the analysis as the participant was unable to complete all experimental tasks due to being heavily pregnant at the time of participation. All participants had normal or corrected-to-normal vision. All participants provided informed consent. This study was approved by the ethics committee at Lancaster University.

#### Stimuli and apparatus

For the optic flow calibration phase, participants walked on a treadmill set to a speed of 2.2 km/h (approximately 0.61 m/s). This treadmill speed was selected as it was reported by participants in our pilot study to be a comfortable speed for one to walk for a prolonged period of time without feeling fatigued. Participants wore an Oculus Rift CV1 head-mounted display (HMD) that displayed a stereoscopic image of the virtual environment with a resolution of 2,160 × 1,200 pixels and a frame rate of 90 Hz. The experimental programme and environment were created using Unity 3D© Gaming Engine, the virtual environment consisted of a horizontal ground plane with grass texture and a brick lane; several 3D models of trees and rock were placed along the path. The 3D camera was placed at eye level, enabling the participant to perceive the virtual environment in a first-person perspective, and the position of the 3D camera was consistent with the participant’s physical eye height. They were positioned in the virtual environment so that they were standing on the brick lane. The movement of the participant’s head was tracked, and graphics were updated as the participant looked around in the virtual environment by moving their head. During the fast optic flow calibration phase, the virtual environment moved past participants at a rate of 6 m/s; during the moderate optic flow calibration phase, the virtual environment moved past participants at a rate of 2 m/s.

**Fig. 1:**
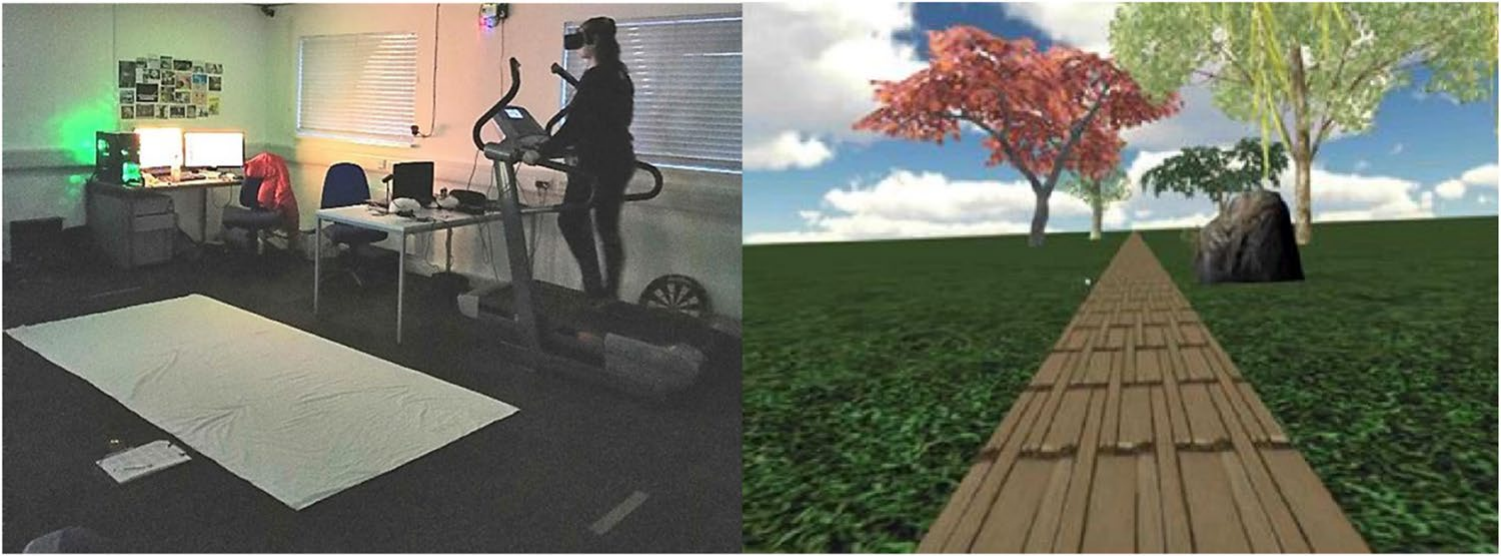
Left panel: Illustration of a participant completing a calibration trial. Right panel: Image of what the participant would see while completing the calibration phase

For the estimation phase, the ground was covered with a sheet of fabric (150 cm × 300 cm). The floor was covered with a piece of white fabric to create a uniform background and minimize landmarks that could influence participants’ judgments (see Fig. 1). A line that served as a reference point was placed 66 cm directly in front of the white fabric, and participants were told to make their estimations while standing behind the line.

#### Procedure

After providing their informed consent, participants were positioned on the treadmill. At the start of the experiment, participants were randomly assigned to experience the moderate optic flow or fast optic flow condition first and were given instructions for both the calibration and estimation phases of the experiment. In the optic flow calibration phase, after donning the Oculus HMD and entering the virtual environment, participants were told to hold onto the treadmill rail and were encouraged to look around to familiarize themselves with the virtual environment before the experiment began. Participants then walked on the treadmill for 10 minutes, while experiencing either the fast or moderate optic flow. After walking for 10 minutes, the experimenter helped the participants remove the HMD, participants were instructed to keep their eyes closed, and they were led off the treadmill and positioned behind the reference line.

The estimation phase consisted of 12 trials, in which participants reported their maximum leaping ability by instructing the experimenter to move the estimation dot (using a laser pointer) closer or farther until it was at the maximum distance the participants believe they could perform a leap from a standing position. A leap is defined as a one-footed takeoff from a standing position and land on the other foot. To control for hysteresis, in half of the trial the estimation dot’s starting position was directly in front of the participants’ feet and at the reference line in front of them; participants moved the dot away from them. For the other half of the trial, the estimation dot’s starting position was at the opposite end of the white fabric covering the floor, at approximately 400 cm away from the participant, and participants moved the dot towards them. Participants were encouraged to make as many adjustments as necessary for an accurate estimation of their leaping ability and to move the dot beyond the area covered by the white fabric if they thought it was necessary, then close their eyes in between trials while the experimenter measured the distance between the reference line and the final location of the estimation dot. After making all 12 estimations and completing the first optic flow condition, participants were led back onto the treadmill and repeated the procedure with the second optic flow condition.

### Results

**Fig. 2:**
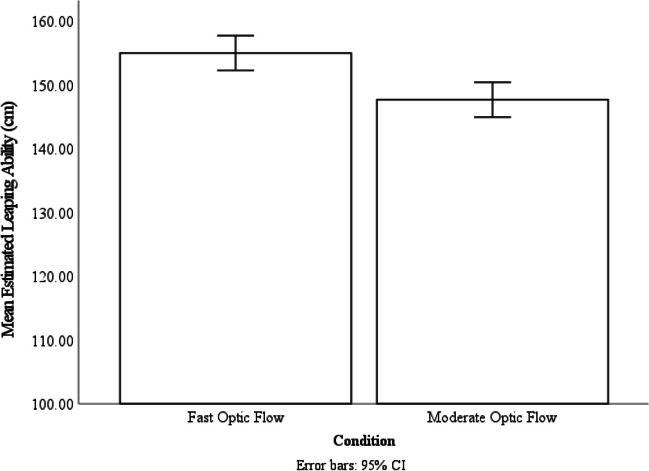
The mean estimated leaping ability of the two optic flow condition. Error bars are 95% CI calculated within subject with the method provided by Loftus and Masson (1994)

To analyze the influence of optic flow calibration on leaping ability estimates, we conducted a repeated-measures ANOVA, with optic flow condition (fast versus moderate) as the within-subjects variable and the estimated leaping ability as the dependent variable. We found a significant effect of optic flow condition on estimated leaping ability, *F*(1, 28) = 7.56, *p* = .01, ƞ_p_^2^ = .21, participants have estimated their leaping ability to be significantly farther in the fast optic flow condition (*M* = 154.94 cm, *SE* = 4.46 cm) than in the moderate optic flow condition (*M* = 147.64 cm, *SE* = 4.04 cm, *p* = .01; see Fig. 2). These results indicated that there was evidence for a difference in leaping-ability estimations between the fast and moderate optic flow conditions, suggesting that optic flow recalibration relative to gait has influenced participants’ subsequent estimations of their leaping ability and participants who experienced fast optic flow estimated their leaping ability more liberally than after they experienced the moderate optic flow.

## Experiment 2

In Experiment 2, we sought to investigate whether recalibrating the relationship between optic flow rate and walking speed could lead to corresponding shifts in judgments of the maximum distance one can jump across with two feet. We expected that the effect of optic flow recalibration to gait would have no or a lesser influence on two-footed jumping, as previous studies have shown that optic flow recalibration to walking would result in no or weak generalization to functionally similar actions that are not as familiar and less well practiced.

### Method

#### Participants

Thirty participants (eight males), 18 to 22 years of age (*M*_age_ = 19.25 years, *SD*_age_ =.92) were recruited from Lancaster University through opportunity sampling. All participants had normal or corrected-to-normal vision. All participants provided informed consent. This study was approved by the ethics committee at Lancaster University.

#### Stimuli and procedure

Everything was the same as in Experiment 1, except participants had to make estimations of their maximum jumping ability (two-footed takeoff and two-footed landing).

### Results

**Fig. 3:**
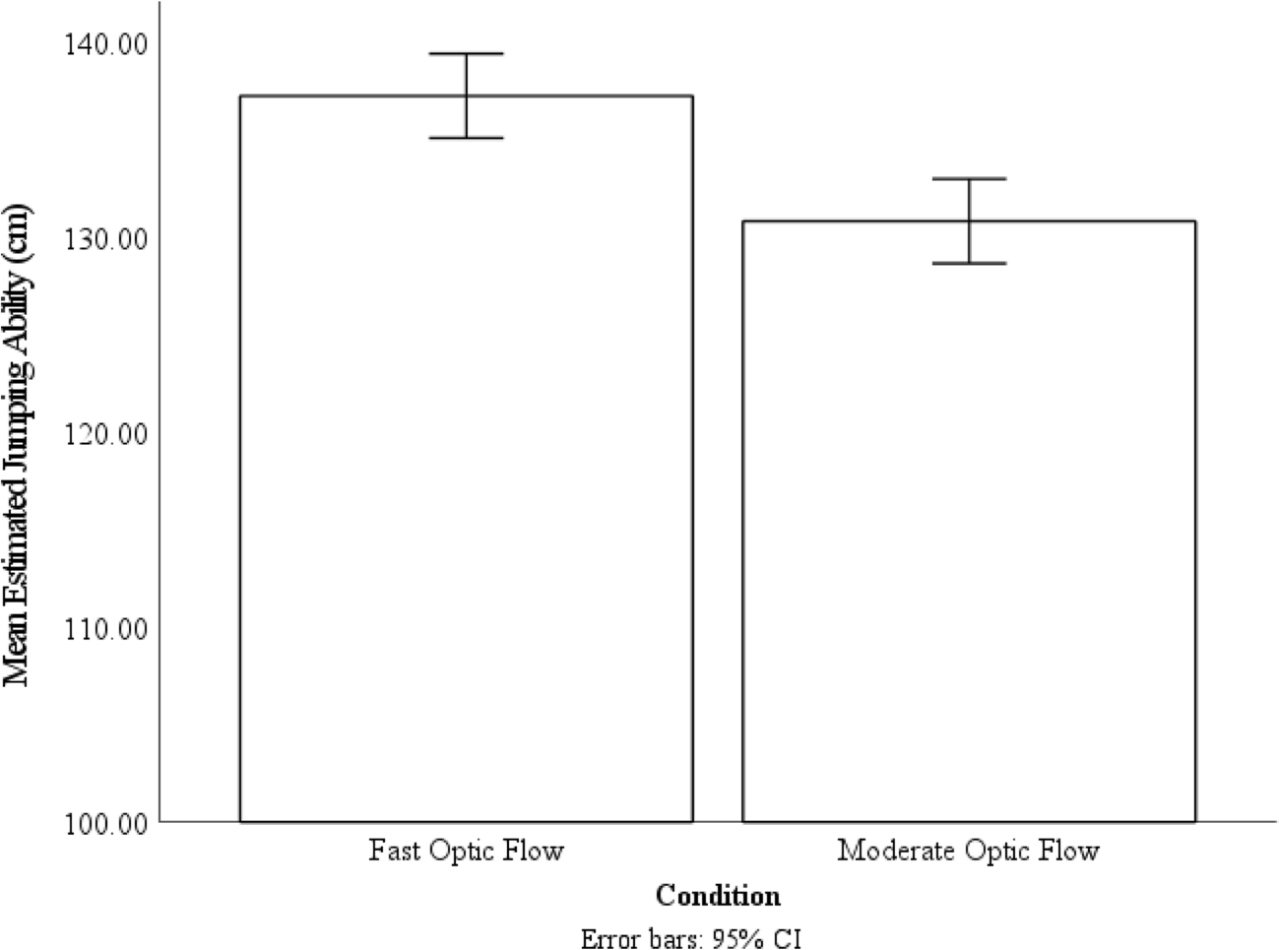
The mean estimated jumping-ability of the two optic flow condition. Error bars are 95% CI calculated within subject with the method provided by Loftus and Masson (1994)

To analyze the influence of optic flow calibration on two-footed jumping ability estimates, we conducted a repeated-measures ANOVA, with optic flow condition (fast versus moderate) as the within-subjects variable and the estimated jumping ability as the dependent variable. We found a significant effect of optic flow condition on estimated jumping ability, *F*(1, 29) = 9.19, *p* = .005, ƞ_p_^2^ = .24, and participants have estimated their jumping ability to be significantly farther in the fast optic flow condition (*M* = 137.26 cm, *SE* = 5.88 cm) than in the moderate optic flow condition (*M* = 130.83 cm, *SE* = 5.59 cm, *p* = .005; see Fig. 3). These results suggested that optic flow speed affects perceived two-footed jumping ability, in which participants who experienced fast optic flow while walking judged that they could jump farther than when they have experienced moderate optic flow while walking. Taken together with the results from Experiment 1, these results suggested that recalibrating the relationship between optic flow rate and walking speed correspondingly shifts judgments of the maximum distance that can be leaped across (an action similar to gait). Moreover, this shift in the action boundary generalizes to the maximum distance that can be jumped across.

### Discussion

An ample body of research has demonstrated people’s ability to accurately determine the locations of their action boundaries for a variety of actions across different environmental contexts. So far, however, limited work has been done to identify the information used to specify action boundaries for launching actions such as leaping and jumping. The current studies aimed to determine whether optic flow calibration relative to gait could influence launching actions such as leaping/jumping. We manipulated perceptual-motor coupling between walking and optic flow speed and calibrated participants to either fast optic flow or moderate optic flow. Participants were asked to estimate the maximum extent of their jumping/leaping ability after being calibrated with the fast or moderate optic flow. Experiment 1 examined how individuals select their action boundaries for leaping after experiencing either fast or moderate optic flow, and Experiment 2 examined how individuals select their action boundaries for jumping following calibration to either fast or moderate optic flow.

We felt the need to point out that, while it is typical in the optic flow recalibration literature to utilize a pretest–calibration–posttest design, in which participants perform a motor task before and after exposure to different optic flow speeds. We have not included such a design, nor have we compared participants’ perceived action capabilities with their actual action capabilities, as these comparisons would only be meaningful if we were interested in individuals’ affordance judgment accuracy. This is not the main question we were addressing, as we were interested in the *relative difference* in estimated action capabilities following exposure to different optic flow speeds relative to gait.

We found that individuals recalibrated to different-sized action boundaries following experience with different optic flow speeds. Participants were more liberal with their estimates and estimated their leaping ability to be significantly farther after experiencing the fast optic flow than when they were calibrated to the moderate optic flow. This finding was consistent across both experiments and provides evidence that optic flow recalibration does influence the perception of action boundary for leaping as well as two-footed jumping. We expected a difference for only leaping because the action was similar to walking, but we still found an effect for two-footed jumping which suggests generalization across actions that require different types of movements.

This finding is interesting, because if this generalization is solely driven by optic flow recalibration relative to gait, exposure to different optic flow speeds relative to gait should have no or little meaningful influence on subsequent two-footed jumping estimates. Previous studies have shown that optic flow recalibration did not, or only weakly, generalize to other locomotor actions functionally similar to walking but less well-practiced, such as single leg hopping and wheelchair wheeling (Durgin et al., 2004; Kunz et al., 2013; Rieser et al., 1995). However, in our study, we found that not only did this recalibration generalize to two-footed jumping, but the magnitude of difference between the two optic flow conditions was similar across both actions. It is plausible that neither leaping nor two-footed horizontal jumping is more common or well-practiced than the other, thus explaining the observed similarities in recalibration effects between the two actions. However, regardless of whether one form of jumping is more prevalent or whether both are equally practiced, our findings suggest that optic flow calibration relative to walking can be generalized to actions involving significantly different movement patterns. Therefore, we speculate that the manipulation of optic flow relative to walking affected one’s anticipated effort required to perform an action. Exposure to fast optic flow relative to gait might lead individuals to perceive actions as less effortful when covering greater distances, while moderate optic flow might result in perceiving greater effort needed for a given distance. This expectation of effort to traverse a prescribed distance could influence one’s perceived action capability for launching actions such as jumping and leaping.

Indeed, a wealth of literature has shown that optic flow manipulation affects anticipated effort. For example, previous studies demonstrated that the manipulation of optic flow relative to gait could influence the perception of travelled distance when a discrepancy was introduced between anticipated patterns of optic flow and walking effort. Proffitt et al. (2003) had participants walk on a treadmill while wearing an HMD that provided them with no optic flow, and during calibration, participants learned from the locomotor experience that it took more effort to remain “optically” stationary. Following calibration, when asked to walk blindly to a target, participants consistently demonstrated an overestimation of the distance to the target. Similarly, another recent study manipulating optic flow speed and anticipated effort has shown its influence on the perceived steepness of hills. Linkenauger and Readman (2020) had participants walk on a treadmill with optic flow that was either faster or slower than the walking speed, and participants learned that it took little effort to traverse a great distance in the fast optic flow condition whereas they learned that despite exerting a great deal of effort, they were unable to traverse very far in the slow optic flow condition. The findings demonstrated that after experiencing slower optic flow relative to gait, participants estimated hills to be steeper than when they experienced the faster optic flow. Taken together, these findings suggest that the manipulation of optic flow relative to gait could influence the anticipated effort to perform a given action, which in turn influences an individual’s perception of spatial properties.

Hence, we suspect that the anticipated effort to perform a given action induced by optic flow recalibration may have played a larger role than just optic flow recalibration to gait alone, and possibly, the anticipated effort resulting from walking and optic flow may serve as the informational basis on which we use to anticipate our abilities for actions that involve very different movements (Franchak, 2020). Nevertheless, this is purely speculative, as we did not directly measure anticipated effort, and we did not set out to fully explain the mechanisms underlying this effect in one paper. Hence, further research is needed to understand the underlying mechanism and explore the generalization of the effect of optic flow recalibration to an action in far space that is less ambulatory.

An alternative explanation might be that the manipulation of optic flow speed relative to gait influences spatial scaling/distance perception, in which the exposure to different optic flow speeds relative to gait has influenced participants’ distance perception. Suppose the visual information in optic flow for distance perception is the difference in time-to-contact (TTC) between the near and far edges of a given extent. This ∆TTC information is used to control walking, stepping, leaping, and two-foot jumping. This faster optic flow condition shifts this relation, such that the same ∆TTC corresponds to a larger extent. Thus, after recalibration, the same ∆TTC is used to control crossing a larger extent, for all actions. Thus, it is possible that the common visual information underlies the shift in the action boundary and generalizes across actions. This explanation is consistent with previous studies that demonstrated optic flow recalibration influences participants’ subsequent distance perception, in that participants tended to underestimate/undershoot or overestimate distance/overshoot following exposure to visually faster or slower optic flow (Adams et al., 2018; Mohler et al., 2007; Ziemer et al., 2013). Nevertheless, this is only speculation as we did not investigate participants’ distance perception, but rather their estimated ability to perform a leap/two-footed jump.

Taken together, the results of our current studies demonstrate that optic flow calibration for walking not only informs the perception of affordances similar to walking but also allows us to anticipate our abilities for actions involving different movement patterns. While it is unlikely for optic flow to be the sole source of perceptual information for scaling jumping and leaping actions, it may play a role in specifying an action boundary when calibrated or scaled to such actions. Previous research has shown that different actions can be scaled based on geometrical properties, dynamic properties, and optical variables available to the perceiver. For instance, eye height is the relevant perceptual metric for scaling the distance to a given target (Cutting & Vishton, 1995), while judgments of stepping-on ability rely on a combination of eye height and leg length (Mark, 1987). Additionally, when combined with shoulder width as well as dynamic information such as stride length, eye height helps determine the width of aperture that affords passing through (Fath & Fajen, 2011; Warren & Whang, 1987).

In sum, our findings provide evidence that optic flow may specify an action boundary when calibrated or scaled to actions such as leaping and jumping. By manipulating optic flow speed, we may potentially influence the anticipated effort required to cover a prescribed distance, which, in turn, could impact an individual's perceived action capabilities for jumping and leaping. However, further research is needed to fully comprehend the mechanisms underlying these effects and how optic flow calibration may generalize to actions in different spatial contexts. Our study contributes to a growing understanding of how perceptual-motor coupling, particularly related to optic flow and walking, shapes individuals’ perceived action capabilities for various motor tasks.
